# The effect of tolvaptan on total kidney volume and inflammatory markers in autosomal dominant polycystic kidney disease

**DOI:** 10.1007/s10157-026-02910-0

**Published:** 2026-06-25

**Authors:** Merve Oruc, Mehmet Burak Ercin, Ahmet Oruc, Kultigin Turkmen, Ismail Baloglu

**Affiliations:** 1 Department of Nephrology, Meram School of Medicine, Necmettin Erbakan University, Konya, Turkey; 2 Department of Medical Oncology, Meram School of Medicine, Necmettin Erbakan University, Konya, Turkey

**Keywords:** Autosomal dominant polycystic kidney disease (ADPKD), Tolvaptan, Total kidney volume, Neutrophil-to-lymphocyte ratio, Systemic immune-inflammation index, Inflammation

## Abstract

**Background:**

Autosomal dominant polycystic kidney disease (ADPKD) is characterized by progressive cyst growth, kidney enlargement, and decline in renal function. Increasing evidence suggests that inflammatory processes may contribute to disease progression. This study aimed to evaluate the association between tolvaptan use, systemic inflammatory markers, and total kidney volume (TKV) progression in patients with ADPKD.

**Methods:**

This retrospective study included 67 patients with ADPKD, including 40 receiving tolvaptan and 27 untreated controls. Neutrophil-to-lymphocyte ratio (NLR), platelet-to-lymphocyte ratio (PLR), systemic immune-inflammation index (SII), estimated glomerular filtration rate (eGFR), and TKV were evaluated at baseline and after 24 months. Longitudinal changes were assessed using Wilcoxon signed-rank tests and linear mixed-effects models.

**Results:**

Patients receiving tolvaptan were younger and had higher baseline TKV values than untreated patients. During follow-up, NLR, PLR, and SII decreased in the tolvaptan group, whereas these markers increased in untreated patients. eGFR declined in both groups but appeared less pronounced in patients receiving tolvaptan. TKV increased in both groups; however, the percentage increase in TKV was lower among patients receiving tolvaptan. Significant time × treatment interactions were observed for NLR, PLR, SII, and TKV. In the non-tolvaptan group, NLR at 24 months showed a significant positive association with TKV.

**Conclusions:**

Tolvaptan use was associated with differences in longitudinal changes in systemic inflammatory markers and kidney volume progression in ADPKD. Further prospective studies are needed to clarify the clinical relevance of these associations.

## Introduction

Autosomal dominant polycystic kidney disease (ADPKD) is a common monogenic kidney disorder characterized by the progressive development of renal cysts and extrarenal manifestations. Most frequently caused by pathogenic variants in *PKD1 and PKD2*. The typical clinical phenotype includes the formation of multiple bilateral renal cysts, progressive kidney enlargement, and gradual decline in renal function [1, 2].

Treatments targeting the effect of arginine vasopressin, an antidiuretic hormone, on the vasopressin-2 receptor found in the epithelial cells that form kidney cysts are the standard treatment method used in ADPKD patients at high risk of rapid disease progression. Tolvaptan, a vasopressin V2 receptor antagonist, is recognized as a disease-modifying treatment in ADPKD and reduces the increase in total kidney volume (TKV). In the TEMPO 3:4 study, tolvaptan was shown to significantly slow the decline in estimated glomerular filtration rate (eGFR). Since the decline in eGFR is a widely accepted surrogate endpoint for assessing the onset of renal failure, these findings suggest that tolvaptan may delay the progression of renal function decline [2–4].

Because ADPKD progresses over time, increasing attention has been directed toward the contribution of inflammatory processes, immune signaling, and the renal microenvironment to cyst enlargement and fibrotic remodeling. Although the immune microenvironment is widely recognized as a key determinant in tumor biology, its relevance in ADPKD has not been examined to the same extent, despite several pathological similarities between cystic disease and tumor-like processes. Evidence from both patients and experimental models consistently demonstrates the presence of chronic inflammatory activity within cystic kidneys, suggesting that immune-related mechanisms participate in regulating cyst expansion while simultaneously contributing to the gradual deterioration of renal function [5, 6].

Interstitial inflammation in ADPKD is associated with cyst expansion, interstitial remodeling, and hypertension. Nevertheless, this phenomenon needs to be considered within the wider context of CKD, in which mechanisms such as interstitial fibrosis, tubular atrophy, and microvascular damage are central drivers of disease progression [7].

Recent evidence indicates that tolvaptan has been reported to be associated with reduced inflammatory activity and slow the increase in total kidney volume in ADPKD, suggesting that its anti-inflammatory effects may contribute to the drug’s disease-modifying benefits [8].

This study aimed to assess the association between tolvaptan use, inflammatory markers, and total kidney volume progression in patients with ADPKD and to explore whether changes in inflammatory indices may be associated with disease progression.

## Methods

### Study design and population

This retrospective study screened 92 patients followed at the Nephrology outpatient clinic of Necmettin Erbakan University Faculty of Medicine, Konya, Türkiye of whom 67 met the inclusion criteria and were included in the analysis. Forty patients receiving tolvaptan constituted the treatment group, while 27 patients who did not receive tolvaptan served as the control group. Among those treated with tolvaptan, the majority (*n* = 34) received the standard 90/30 mg split-dose regimen, whereas 6 patients received reduced doses (45/15 mg) according to their eGFR and drug tolerability.

### Inclusion and exclusion criteria

**Inclusion criteria** consisted of adult patients diagnosed with ADPKD according to established radiologic criteria (bilateral renal cysts detected on imaging). Patients were required to have at least two magnetic resonance imaging (MRI) examinations performed at two-year intervals including T2-weighted sequences, as well as available laboratory data including serum creatinine, estimated glomerular filtration rate (eGFR), and complete blood counts for calculation of neutrophil-to-lymphocyte ratio (NLR), platelet-to-lymphocyte ratio (PLR), and systemic immune-inflammation index (SII).

For the tolvaptan treatment group, patients were required to meet the reimbursement criteria defined by the Turkish Health Practice Communiqué (SUT). Accordingly, eligible patients were between 18 and 55 years of age and had an eGFR > 25 mL/min/1.73 m². In addition, patients were required to demonstrate rapid disease progression, defined as either:

1. A mean annual reduction in eGFR of ≥ 3 mL/min/1.73 m² based on a minimum of five measurements obtained over a follow-up period of at least four years, or.
2. Classification as Mayo Imaging Class 1D or 1E based on kidney volume measurement by MRI or CT.

Age-specific eGFR criteria recommended by SUT were also considered in treatment eligibility:

- 18–39 years: no eGFR threshold required.
- 40–44 years: eGFR < 90 mL/min/1.73 m².
- 45–49 years: eGFR < 75 mL/min/1.73 m².
- 50–55 years: eGFR < 60 mL/min/1.73 m².

**Exclusion criteria** included history of kidney transplantation or nephrectomy, concomitant chronic kidney diseases other than ADPKD, active infections, autoimmune disorders, or malignancies at the time of laboratory evaluation, incomplete MRI or laboratory data, pregnancy or breastfeeding, and patients with eGFR < 25 mL/min/1.73 m², in accordance with SUT regulations for discontinuation of tolvaptan therapy [9].

Active infection was defined based on available clinical documentation, including recorded symptoms, signs of infection, or ongoing antibiotic use at the time of evaluation. Patients with documented evidence of acute infection were excluded. CRP levels were not applied as a strict exclusion criterion; however, patients with documented clinical evidence of acute inflammation or infection were not included. Chronic low-grade inflammatory states were not specifically excluded, as they are considered part of the disease process in ADPKD.

### Kidney volume assessment

Total kidney volumes were assessed using MRI performed at two-year intervals in the T2-weighted sequence. Kidney volume was calculated using the ellipsoid formula:

Kidney Volume= (π/6) × length × width × depth, where length, width and depth correspond to the craniocaudal, mediolateral, and anteroposterior dimensions of the kidney, respectively [10].

Although volumetric segmentation methods may provide more precise measurements, the ellipsoid formula is widely used in clinical studies and provides reliable estimates of kidney volume.

In addition to absolute TKV values, the relative percentage change in total kidney volume over the follow-up period was calculated to better assess kidney growth. The percentage change in TKV was calculated using the following formula: TKV change (%) = (TKV at 24 months − TKV at baseline)/TKV at baseline × 100. All MRI measurements were performed by an experienced radiologist blinded to the treatment groups to minimize measurement bias.

### Laboratory measurement

Laboratory parameters, including PLR, NLR, SII, serum creatinine and eGFR were recorded. SII was calculated as platelet count × neutrophil count/lymphocyte count.

### Ethics statement

The study protocol was approved by the Medical Ethics Committee of Necmettin Erbakan University (Konya, Turkey).

Ethical Approval Number :6399.

### Statistical analysis

Statistical analyses were performed using IBM SPSS Statistics version 25.0. Continuous variables were summarized as median (interquartile range, IQR) or mean ± standard deviation according to data distribution. Categorical variables were summarized as frequencies and percentages. Baseline comparisons between groups were performed using the Mann–Whitney U test for continuous variables and the Chi-square test for categorical variables. Unadjusted within-group longitudinal changes from baseline to month 24 were analyzed using the Wilcoxon signed-rank test. To further evaluate longitudinal changes while accounting for repeated measurements and potential baseline confounding, adjusted linear mixed-effects models were performed including time, treatment group, time × treatment interaction, age, baseline eGFR, baseline TKV, and sex as fixed effects. Patient ID was included as a random intercept. Model results were reported as estimated β coefficients, standard errors (SE), 95% confidence intervals (CI), and p values. A two-sided p value < 0.05 was considered statistically significant.

## Results

A total of 67 patients with ADPKD were included in the study, including 40 patients treated with tolvaptan and 27 patients who did not receive tolvaptan. Patients receiving tolvaptan were significantly younger than untreated patients (*p* = 0.007) and had significantly higher baseline TKV values (*p* = 0.009). Younger age categories were more frequently observed in the tolvaptan-treated group, with 55.0% of treated patients aged < 43 years compared with 29.6% in the untreated group (*p* = 0.041). In addition, patients receiving tolvaptan were more commonly classified as Mayo imaging class D/E, whereas untreated patients were more frequently classified as Mayo class A–C (87.5% vs. 44.4%; *p* < 0.001). Baseline inflammatory markers, including NLR, PLR, and SII, were comparable between the groups (all *p* > 0.05) (Table 1).

**Table 1:** Baseline demographic, clinical, and inflammatory characteristics of patients with ADPKD according to tolvaptan treatment

Variable	Tolvaptan (+) (*n* = 40)	Tolvaptan (−) (*n* = 27)	*p* value
**Age (years)**,** median (IQR)**	40.5 (32.8–49.0)	48.0 (42.0–54.0)	0.007*
Age < 43 years, n (%)	22 (55.0)	8 (29.6)	0.041*
Age ≥ 43 years, n (%)	18 (45.0)	19 (70.4)	
**Sex (M/F)**,** n (%)**	24 (60)/16 (40)	15 (55.6)/12 (44.4)	0.803
**BMI (kg/m**^**2)**^, **median (IQR)**	26.3 (24.0–30.7)	27.3 (24.5–30.4)	0.858
**Baseline eGFR (mL/min/1.73 m²)**,** median (IQR)**	60 (36.8–86.5)	72.5 (34.3–94.3)	0.707
**Baseline TKV (ml)**,** median (IQR)**	1899.5 (1174–3410.3)	1058 (644–1980)	0.009*
**Follow-up TKV (ml)**,** median (IQR)**	2121 (1401.5–3388)	1295 (764.3–1903.8)	0.017*
Mayo class A–C, n (%)	5 (12.5)	15 (55.6)	< 0.001*
Mayo class D/E, n (%)	35 (87.5)	12 (44.4)	
**Baseline NLR**,** median (IQR)**	2.31 (1.77–2.79)	2.16 (1.67–2.64)	0.531
**Baseline PLR**,** median (IQR)**	132.15 (93.25–154.6)	140.1 (107.6–158.9)	0.720
**Baseline SII**,** median (IQR)**	638.8 (402.9–816.2)	583.9 (436.7–786.2)	0.734

*ADPKD* autosomal dominant polycystic kidney disease, *BMI* body mass index, *eGFR* estimated glomerular filtration rate, *TKV* total kidney volume, *NLR* neutrophil-to-lymphocyte ratio, *PLR* platelet-to-lymphocyte ratio, *SII* systemic immune-inflammation index, * *p* < 0.05

To further evaluate factors associated with treatment allocation and potential confounding by indication, univariate and multivariable logistic regression analyses were performed (Table 2). In univariate analyses, younger age (*p* = 0.044), lower baseline eGFR (*p* = 0.010), and more advanced Mayo imaging classification (*p* < 0.001) were significantly associated with tolvaptan treatment allocation. In multivariable analyses, younger age (OR 13.8, 95% CI 1.1–174; *p* = 0.041), lower baseline eGFR (OR 0.04, 95% CI 0.004–0.48; *p* = 0.010), and Mayo imaging class D/E (OR 12.3, 95% CI 1.57–96.7; *p* = 0.017) remained independently associated with receiving tolvaptan. Baseline inflammatory indices, including NLR, PLR, and SII, were not independently associated with treatment allocation.

**Table 2:** Univariate and multivariable logistic regression analyses of factors associated with tolvaptan treatment allocation

Variable	Univariate logistic regression Odds ratio (95% CI; *p* value)	Multivariable logistic regression Odds ratio (95% CI; *p* value)
Sex (Male vs. Female)	1.2 (0.44–3.22; 0.718)	–
Age (< 43 vs. ≥ 43 years)	2.9 (1.03–8.16; 0.044)	13.8 (1.1–174; 0.041)
Baseline eGFR (≥ 65 vs. < 65 mL/min/1.73 m²)	0.25 (0.08–0.71; 0.010)	0.04 (0.004–0.48; 0.010)
Baseline TKV (≥ 1650 vs. < 1650 mL)	1.96 (0.73–5.30; 0.181)	2.07 (0.29–14.65; 0.466)
Mayo class (D/E vs. A–C)	8.7 (2.62–29.2; <0.001)	12.3 (1.57–96.7; 0.017)
NLR (≥ 2.16 vs. < 2.16)	1.37 (0.45–4.16; 0.574)	–
PLR (≥ 130.9 vs. < 130.9)	1.37 (0.45–4.16; 0.574)	–

Variables significantly associated with tolvaptan treatment allocation in univariate analyses were entered into the multivariable logistic regression model

*eGFR* estimated glomerular filtration rate, *TKV* total kidney volume, *NLR* neutrophil-to-lymphocyte ratio, *PLR* platelet-to-lymphocyte ratio, *SII* systemic immune-inflammation index, *CI* confidence interval

In unadjusted analyses, inflammatory markers demonstrated different longitudinal patterns according to tolvaptan use over the 24-month follow-up period. In patients receiving tolvaptan, NLR, PLR, and SII significantly decreased during follow-up (*p* = 0.035, *p* = 0.022, and *p* = 0.025, respectively), whereas these markers significantly increased in untreated patients (*p* = 0.019, *p* = 0.037, and *p* = 0.031, respectively). Longitudinal changes in renal function according to tolvaptan use were also evaluated over the 24-month follow-up period. eGFR significantly declined in both groups during follow-up. However, the decline appeared less pronounced in patients receiving tolvaptan compared with untreated patients. These findings provide additional context regarding renal function trajectories alongside the observed changes in inflammatory markers and kidney volume. (Table 3). CRP-to-albumin ratio did not show a significant longitudinal change in either group (all *p* > 0.05).

**Table 3:** Unadjusted 24-month changes in inflammatory markers and total kidney volume in patients with ADPKD according to tolvaptan use

Parameter	Group	Baseline Value (Median, IQR)	24-Month Value (Median, IQR)	*p* value
**NLR**	Tolvaptan +	2.31 (1.77–2.79)	1.89 (1.36–2.40)	0.035*
	Tolvaptan −	2.16 (1.67–2.64)	2.74 (1.61–3.57)	0.019*
**PLR**	Tolvaptan +	132.15 (93.25–154.6)	120 (83.1–148.8)	0.022*
	Tolvaptan −	140.1 (107.6–158.9)	149.3 (126.8–157.5)	0.037*
**SII**	Tolvaptan +	638.8 (402.9–816.2)	522.7 (328–741.7)	0.025*
	Tolvaptan −	583.9 (436.7–786.2)	686.2 (453.7–766.5)	0.031*
**TKV (ml)**	Tolvaptan +	1899.5 (1174–3410.25)	2121 (1401.5–3388)	0.093
	Tolvaptan −	1058 (644–1980)	1765.1 (764.25–1903.75)	0.030*
**eGFR (mL/min/1.73 m²)**	Tolvaptan +	60 (36.75–86.5)	50.6 (29.5–68.5)	0.036*
	Tolvaptan −	72.5 (34.25–94.25)	57.5 (25–82)	< 0.001*

*NLR* neutrophil-to-lymphocyte ratio, *PLR* platelet-to-lymphocyte ratio, *SII* systemic immune-inflammation index, *TKV* total kidney volume, *eGFR* estimated glomerular filtration rate, * *p* < 0.05

**Fig. 1:**
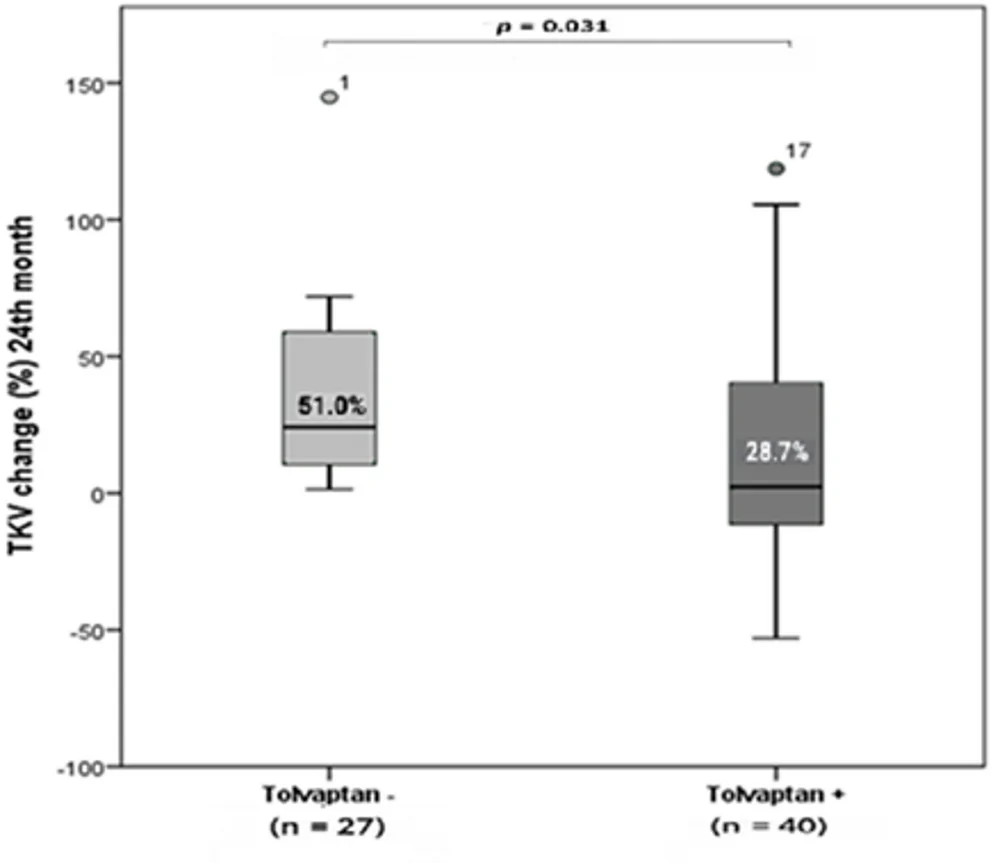
Boxplot showing the percentage change in total kidney volume (TKV) over 24 months according to tolvaptan use. Distinct grayscale tones were applied to improve visualization of treatment groups. Boxes represent the interquartile range, the horizontal line indicates the median, and whiskers represent the minimum and maximum values. Circles indicate outliers

Total kidney volume increased in both groups during follow-up; however, the increase was less pronounced in patients receiving tolvaptan. A statistically significant increase in TKV was observed only in the non-tolvaptan group (*p* = 0.030), whereas the increase in the tolvaptan group did not reach statistical significance (*p* = 0.093). In addition, the percentage increase in TKV over 24 months was significantly lower in patients receiving tolvaptan compared with untreated patients (*p* = 0.031) (Fig. 1).

To further evaluate longitudinal changes while accounting for repeated measurements and potential baseline confounding, adjusted linear mixed-effects models were performed including time, treatment group, time × treatment interaction, baseline age, baseline eGFR, baseline TKV, and sex as fixed effects. Significant time × treatment interactions remained evident for NLR (β = −1.47, *p* = 0.001), PLR (β = −47.4, *p* = 0.022), SII (β = −376.8, *p* = 0.002), and TKV (β = −643.2, *p* = 0.030), indicating that temporal changes differed according to tolvaptan use after adjustment for baseline characteristics. Younger age was independently associated with lower NLR (β = −0.04, *p* < 0.001), PLR (β = −1.6, *p* = 0.003), and SII (β = −10.1, *p* = 0.002). Baseline TKV, baseline eGFR, and sex were not consistently associated with longitudinal inflammatory marker trajectories across models (all *p* > 0.05) (Table 4). Overall, different longitudinal inflammatory marker trajectories and kidney volume progression patterns were observed according to tolvaptan use after adjustment for baseline characteristics.

**Table 4:** Adjusted linear mixed-effects analyses of longitudinal changes in inflammatory markers and total kidney volume over 24 months according to tolvaptan treatment

Outcome	Fixed effect	β	SE	Lower CI	Upper CI	*p* value
**NLR**	Time	0.26	0.22	−0.17	0.81	0.236
	Tolvaptan	1.74	0.37	0.98	2.51	**< 0.001**
	Baseline TKV	−4.77E − 5	5.69E − 5	−6.55E − 5	1.00E − 5	0.404
	Age	−0.04	0.009	−0.061	−0.021	**< 0.001**
	Baseline eGFR	−0.36	0.29	−0.94	0.22	0.219
	Sex	−0.11	0.20	−0.51	0.28	0.560
	Time × Tolvaptan	−1.47	0.41	−2.30	−0.64	**0.001**
**PLR**	Time	10.8	11.3	−11.7	33.5	0.342
	Tolvaptan	66.5	16.7	32.6	100.4	**< 0.001**
	Baseline TKV	−0.002	11.3	−0.009	0.003	0.378
	Age	−1.6	0.55	−2.78	−0.58	**0.003**
	Baseline eGFR	10.2	17.0	−23.7	44.2	0.551
	Sex	−15.1	11.6	−38.3	8.07	0.198
	Time × Tolvaptan	−47.4	10.3	−88.0	−6.8	**0.022**
**SII**	Time	117.2	66.1	−14.4	248.9	0.080
	Tolvaptan	409.4	90.4	225.5	593.3	**< 0.001**
	Baseline TKV	0.01	0.01	−0.02	0.05	0.411
	Age	−10.1	3.1	−16.4	−3.8	**0.002**
	Baseline eGFR	71.0	99.0	−126.0	268.2	0.475
	Sex	−121.5	67.5	−255.9	12.8	0.076
	Time × Tolvaptan	−376.8	115.5	−607.0	−146.7	**0.002**
**TKV (ml)**	Time	46.7	206.9	−366.8	460.3	0.822
	Tolvaptan	−578.0	447.7	−1470.0	314.0	0.201
	Time × Tolvaptan	−643.2	289.4	−1221.4	−65.0	0.030

*NLR* neutrophil-to-lymphocyte ratio, *PLR* platelet-to-lymphocyte ratio, *SII* systemic immune-inflammation index, *TKV* total kidney volume, *eGFR* estimated glomerular filtration rate, *SE* standard error, *CI* confidence interval

**Fig. 2:**
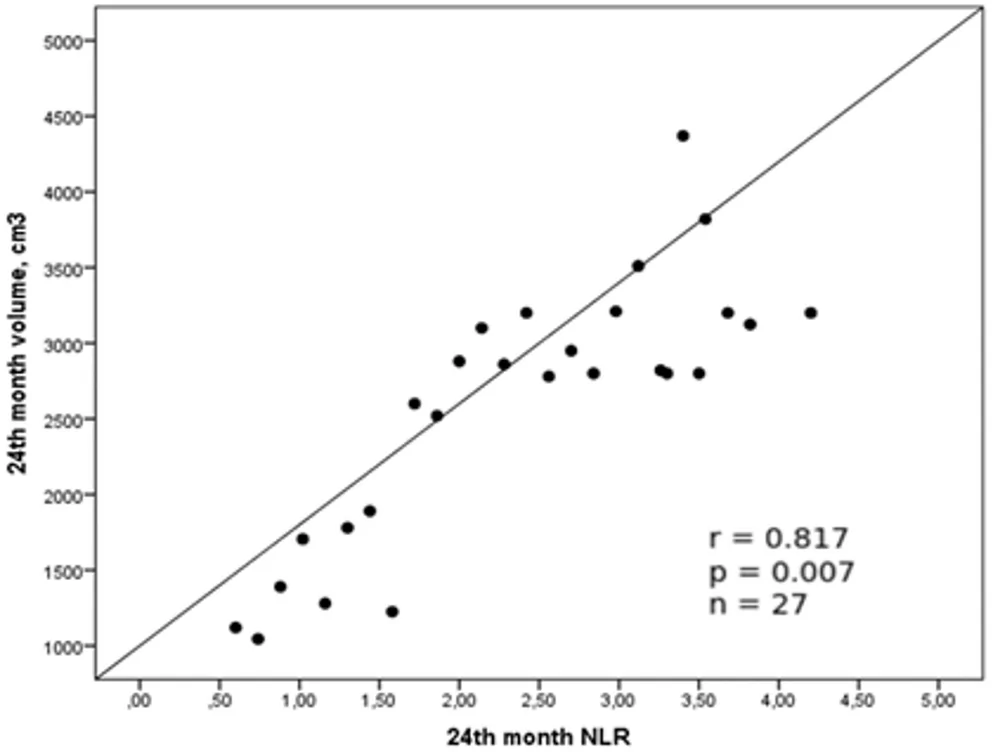
Scatter plot illustrating the correlation between neutrophil-to-lymphocyte ratio (NLR) at 24 months and total kidney volume at 24 months in patients with ADPKD. Each point represents an individual patient. The solid line represents the linear regression line

Correlation analyses demonstrated no significant association between NLR and kidney volume parameters in the tolvaptan-treated group (all *p* > 0.05). In contrast, in the non-tolvaptan group, NLR at 24 months showed a significant positive correlation with TKV at 24 months (*r* = 0.817, *p* = 0.007) (Fig. 2).

## Discussion

Tolvaptan has been shown to slow disease progression in ADPKD by reducing the rate of eGFR decline and TKV growth. In large randomized trials such as TEMPO 3:4 and REPRISE, tolvaptan significantly reduced TKV increase and slowed renal function decline compared with placebo [2, 11, 12]. Real-world studies have reported similar benefits, although the effect on TKV growth appears more variable [13–15]. Consistent with these findings, TKV increased in both groups in our study, but the increase reached statistical significance only in patients not receiving tolvaptan.

While inflammation is not regarded as a primary determinant of cyst formation, its contribution to disease progression, along with the involvement of alternative activation pathways, is increasingly acknowledged. Previous studies have explored the relationship between inflammatory mediators and disease severity in ADPKD. Analyses based on both continuous and grouped approaches have demonstrated that higher levels of pro-inflammatory cytokines such as IL-6 and TNF-α are significantly associated with increased htTKV and a higher risk of disease progression [16].

Potential anti-inflammatory properties of tolvaptan have been suggested in various preclinical disease models. Experimental studies have reported that tolvaptan reduces inflammatory cell infiltration and pro-inflammatory cytokine expression, and may also modulate processes associated with oxidative stress. Interstitial inflammation plays a role in cyst progression in ADPKD, with macrophage infiltration as a key component of the inflammatory microenvironment [17, 18]. Studies in animal models have shown that tolvaptan inhibits cellular proliferation and cyst development by reducing vasopressin-mediated cAMP production in the kidney. Additionally, it has been proposed that the reduction in inflammatory cytokine production may limit tissue damage, thereby slowing the rate of TKV progression, and that decreased levels of lipocalin associated with neutrophil gelatinase a key biomarker of kidney damage may slow the progression of renal injury [19]. In this context, the observed changes in inflammatory indices in our study may reflect alterations in systemic inflammatory processes associated with tolvaptan use.

Tolvaptan treatment has also been associated with significant reductions in inflammatory markers such as CRP, SII, NLR, and PLR, accompanied by a more stable course of renal function [8]. In contrast, untreated patients exhibited increases in these markers along with a more pronounced decline in eGFR. Similar trends have also been described in ADPKD populations, where systemic inflammatory markers have been linked to adverse vascular and metabolic profiles [8, 20]. In our study, we observed a decrease in NLR, PLR, and SII levels in patients receiving tolvaptan, whereas these markers increased in the control group, supporting the overall pattern reported in the literature. In addition to unadjusted analyses, adjusted longitudinal mixed-effects models including baseline age, baseline eGFR, baseline TKV, and sex demonstrated significant time × treatment interactions for inflammatory markers and TKV, suggesting that temporal trajectories differed according to tolvaptan use after accounting for major baseline characteristics. However, in contrast to previous reports, CRP-alb levels did not show a significant change in either group in our cohort. To further explore potential confounding by indication, additional logistic regression analyses were performed to evaluate factors associated with treatment allocation. Younger age and more advanced Mayo imaging class were independently associated with receiving tolvaptan, reflecting expected clinical treatment selection patterns. These findings support the presence of baseline differences between treatment groups and emphasize the importance of adjusted longitudinal analyses when interpreting treatment-related associations.

This discrepancy may suggest that different inflammatory markers reflect distinct aspects of systemic inflammation. Hematological indices such as SII and related parameters may not correlate directly with CRP, particularly in the context of low-grade inflammation [21]. In this regard, the observed changes in NLR, PLR, and SII in our study may reflect alterations in low-grade systemic inflammation or immune cell dynamics rather than acute-phase responses. These findings are consistent with the growing use of hematological inflammatory indices such as NLR and PLR as markers of systemic inflammation in chronic kidney disease populations.

This distinction may be particularly relevant in ADPKD, where chronic inflammatory mechanisms are thought to contribute to disease progression. Nevertheless, these indices remain nonspecific and should be interpreted with caution, and given the observational design, the findings should be considered as associations rather than evidence of a causal effect.

These findings suggest that tolvaptan use may be associated with differences in systemic inflammatory marker trajectories in ADPKD. However, these observations should be interpreted as associations rather than evidence of an independent causal effect.

In our study, the increase in TKV during the follow-up period was statistically significant only in patients not receiving tolvaptan. This observation remained directionally consistent in adjusted longitudinal analyses. Another noteworthy finding was the strong positive correlation observed between NLR and kidney volume in the group not receiving tolvaptan. This observation may support a potential relationship between systemic inflammatory activity and kidney volume progression in ADPKD. However, this finding should be interpreted cautiously given the relatively small subgroup size and exploratory nature of the analysis. In addition, the observed association may be influenced by baseline disease severity differences or potential outliers and therefore requires confirmation in larger prospective studies. The absence of a similar correlation in the tolvaptan-treated group may suggest differences in inflammatory marker trajectories according to treatment status; however, causal interpretations cannot be made based on the present observational design.

However, it has been reported that tolvaptan treatment may be associated with a risk of hepatotoxicity [22]. Therefore, regular and careful monitoring of liver function is recommended, especially in patients receiving long-term treatment. Although tolvaptan’s potential anti-inflammatory and nephroprotective effects appear promising, the balance between the clinical benefits it provides and the potential risks must be carefully evaluated when planning treatment.

## Limitations

This study is subject to certain limitations. In particular, the retrospective design and the relatively limited sample size may reduce the generalizability of the results. Additionally, not all patients received the same dose of tolvaptan, as some were treated with reduced doses based on their eGFR and drug tolerability. Furthermore, the duration of tolvaptan therapy prior to study inclusion was not uniformly recorded, although most patients received the standard daily dose of 120 mg and had been treated for at least two years. Second, total kidney volume was calculated using the ellipsoid formula based on MRI measurements rather than volumetric segmentation, which may provide more precise assessments. Patients receiving tolvaptan had larger baseline TKV values and more advanced Mayo imaging classifications, reflecting expected clinical treatment selection patterns. Therefore, differences observed during follow-up should be interpreted in the context of baseline disease severity. Although adjusted longitudinal models were performed including major baseline covariates, residual confounding due to unmeasured disease severity factors and treatment-selection bias cannot be completely excluded. Although CRP-alb was used to assess acute-phase inflammation, it may not fully exclude all potential confounding factors. In addition, NLR, PLR, and SII are influenced by multiple clinical conditions and may not specifically reflect kidney-related inflammatory processes. Inflammatory activity was evaluated using systemic inflammatory markers rather than direct assessment of renal inflammatory processes. Given the retrospective design and non-randomized treatment allocation, causal relationships cannot be established. Future prospective studies with larger populations and longer follow-up are needed to further clarify these relationships.

## Conclusion

In conclusion, this study suggests that tolvaptan use may be associated with a slower progression of renal volume increase in patients with ADPKD, along with favorable changes in systemic inflammatory markers. Overall, our findings suggest that changes in hematological inflammatory indices may be associated with tolvaptan use and with differences in TKV progression in ADPKD. These findings suggest that differences in inflammatory marker trajectories may accompany tolvaptan use and disease progression in ADPKD; however, the underlying mechanisms and clinical implications require further investigation.

From a clinical perspective, these readily available markers may offer additional insight into systemic inflammatory status and disease progression; however, their clinical utility remains to be clarified. Further prospective studies with larger patient populations and longer follow-up are needed to better define the relationships between inflammation, kidney volume progression, and the therapeutic effects of tolvaptan in ADPKD.
